# Synthesis and Mechanistic Study of a Copolymer Demulsifier for Dehydration of Water‐in‐Oil Emulsion of Crude Oil

**DOI:** 10.1002/open.202500188

**Published:** 2025-06-25

**Authors:** Xuezhi Li, Bin Ma, Liming Fu, Jing Bai, Qingbing Zhang, Baolu Yu

**Affiliations:** ^1^ China National Petroleum Corporation The 3rd oil production plant of Qinghai Oilfield Branch Haixi Mongol Tibetan Autonomous Prefecture Qinghai 816400 China

**Keywords:** copolymers, dehydration, demulsifiers, water‐in‐oil emulsion of crude oil

## Abstract

This study presents the synthesis and characterization of a novel polymeric demulsifier, P(AM‐EHMA‐VBS‐VP), through emulsion polymerization for efficient separation of water‐in‐crude oil emulsions. The synthesis parameters are systematically optimized using orthogonal array design complemented by single‐factor experiments. The demulsification performance is evaluated under simulated field conditions, with particular emphasis on dosage optimization and temperature effects. Comprehensive mechanistic investigations are conducted through dynamic interfacial tension measurements, interfacial dilational rheology analysis, and zeta potential characterization to elucidate the demulsification mechanism and the impact of inorganic salts on demulsification efficiency. The optimized synthesis conditions yield a copolymer with monomer mass ratios of AM:EHMA:VBS:VP = 1:4:4:1, achieved at 60 °C for 8 h with 30% monomer concentration and 0.15% initiator dosage. Optimal demulsification performance is observed at 80 °C with a demulsifier concentration of 300 mg L^−1^. The synthesized demulsifier demonstrates remarkable salt tolerance, maintaining effectiveness in environments containing up to 30 000 mg L^−1^ NaCl and 10 000 mg L^−1^ CaCl_2_. Mechanistic studies reveal that the demulsifier operates through interfacial adsorption, which simultaneously reduces the mechanical strength of the interfacial film and decreases the surface charge density of emulsion droplets. This dual mechanism effectively compromises the emulsion stability by diminishing both the film's resistance to deformation and the electrostatic repulsion between droplets.

## Graphical abstract

P(AM‐EHMA‐VBS‐VP), a novel polymeric demulsifier, adsorbs at the interface, reduces interfacial modulus, overcomes asphaltene‐inhibited coalescence, and promotes droplet coalescence for effective demulsification.

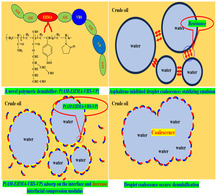

## 1 Introduction

Crude oil, as a pivotal global energy resource, plays a critical role in maintaining energy security and economic stability.^[^ ^1^, ^2^, ^3^ ^]^ The extraction and processing of crude oil, however, are frequently complicated by the formation of water‐in‐oil (W/O) emulsions, a phenomenon resulting from the complex geological conditions and the presence of natural surfactants during oil recovery.^[^ ^4^, ^5^, ^6^, ^7^ ^]^ These emulsions, formed through mechanical shearing and mixing during extraction processes, present significant challenges to oil transportation, refining operations, and overall process efficiency.^[^ ^8^, ^9^ ^]^ The economic implications are substantial, as elevated water content not only diminishes the market value of crude oil but also escalates operational costs associated with water transportation and disposal.^[^ ^10^, ^11^, ^12^, ^13^ ^]^

Current demulsification strategies in oilfield operations primarily encompass physical, chemical, and biological methods.^[^ ^14^, ^15^, ^16^ ^]^ Among these, chemical demulsification has emerged as the most prevalent approach due to its operational simplicity and effectiveness.^[^ ^14^ ^]^ This technique involves the addition of surface‐active agents that migrate to the oil–water interface, reducing interfacial tension and surface charge density, thereby destabilizing the protective film around water droplets and facilitating coalescence.^[^ ^17^, ^18^, ^19^ ^]^ However, the efficacy of chemical demulsification is influenced by multiple factors, including crude oil properties (viscosity, wax content, and asphaltene concentration), demulsifier characteristics (molecular structure, hydrophilic‐lipophilic balance value, and dosage), and operational parameters (temperature, pressure, and mixing intensity).^[^ ^20^, ^21^, ^22^, ^23^, ^24^ ^]^ Particularly challenging are high‐viscosity crude oils with elevated wax content, which require tailored demulsification strategies.

The chemical demulsifier market is currently dominated by nonionic block polyether compounds,^[^ ^25^, ^26^, ^27^ ^]^ typically synthesized through anionic ring‐opening polymerization of ethylene oxide (EO) and propylene oxide (PO) using various initiators. These amphiphilic molecules effectively migrate to oil–water interfaces, displacing natural surfactants and destabilizing the interfacial film.^[^ ^27^ ^]^ However, the synthesis of these compounds presents significant safety concerns due to the flammable nature of EO and PO monomers and the requirement for high‐temperature, high‐pressure reaction conditions.^[^ ^28^ ^]^ This has spurred research into alternative demulsifier chemistries, particularly amphiphilic polymers synthesized through copolymerization of acrylate derivatives with various vinyl monomers,^[^ ^29^, ^30^ ^]^ which offer safer synthesis routes and comparable performance.

In this context, we report the development of a novel polymeric demulsifier through emulsion copolymerization of acrylamide (AM), 2‐ethylhexyl methacrylate (EHMA), sodium 4‐vinylbenzenesulfonate (VBS), and vinylpyrrolidone (VP). Herein, the monomer AM exhibits excellent water solubility and high polymerization degree, which can effectively increase the molecular weight of the polymeric surfactant. The EHMA monomer contains an iso‐octyl ester structure that inhibits intermolecular chain association, thereby reducing the viscoelasticity of the copolymer solution. Consequently, this effectively decreases the elastic modulus of the copolymer at the oil–water interface, weakening emulsion stability. As a result, the synthesized copolymer demonstrates both high interfacial activity and reduced interfacial compression modulus. The VBS monomer incorporates a sulfonic acid group and a rigid benzene ring structure, significantly enhancing the copolymer's salt resistance while increasing the rigidity of the molecular chains to prevent high viscoelasticity in aqueous solution. Additionally, the VP monomer possesses a moderately rigid structure, which assists VBS in regulating the rigidity characteristics of the copolymer molecular chains, thereby improving the demulsification efficiency of the copolymer. The synthesis process was systematically optimized, and demulsification conditions were carefully investigated. Furthermore, we employed advanced characterization techniques, including dynamic interfacial tension measurements, zeta potential analysis, and interfacial dilational rheology, to elucidate the demulsification mechanism and evaluate the impact of inorganic salts on demulsification efficiency.

## 2 Experimental Section

### 2.1 Materials

AM, EHMA, VBS, and VP, analytical reagent (AR) grade, were purchased from Shanghai McLean Biochemical Technology Co., Ltd. Sodium dodecyl benzene sulfonate (SDBS), nonylphenyl‐polyethylene glycol solution (NP‐10), AR grade, were obtained from Shanghai Aladdin Biochemical Technology Co., Ltd. Magnesium chloride, sodium chloride, calcium chloride, ammonium persulfate, sodium sulfite were purchased from Chengdu Cologne Chemical Reagent Co., Ltd. KBr, deuterium‐oxide, and HPLC grade were purchased from Shanghai Aladdin Biochemical Technology Co., Ltd. The crude oil was obtained through on‐site sampling from the oil production plant of Yumen oilfield.

All chemicals were used as received without further purification. Acrylamide (AM, AR grade), 2‐ethylhexyl methacrylate (EHMA, AR grade), sodium 4‐vinylbenzenesulfonate (VBS, AR grade), and vinylpyrrolidone (VP, AR grade) were procured from Shanghai McLean Biochemical Technology Co., Ltd. Sodium dodecyl benzene sulfonate (SDBS, AR grade) and nonylphenyl‐polyethylene glycol solution (NP‐10, AR grade) were obtained from Shanghai Aladdin Biochemical Technology Co., Ltd. Inorganic salts, including magnesium chloride, sodium chloride, and calcium chloride, along with polymerization initiators (ammonium persulfate and sodium sulfite, AR grade), were purchased from Chengdu Cologne Chemical Reagent Co., Ltd. Potassium bromide (KBr, HPLC grade) and deuterium oxide (D_2_O, HPLC grade) for spectroscopic analysis were acquired from Shanghai Aladdin Biochemical Technology Co., Ltd. The crude oil samples were collected from the production facility of Yumen Oilfield (China) and were used as received to maintain the authentic field conditions. The properties of crude oil are shown in **Table** **1**.

**Table 1:** Properties of the medium crude oil sample obtained from the oil production plant.

Density [g cm^−3^]	Wax content [%]	Colloid content [%]	Asphaltene content [%]	Wax precipitation point [°C]	Freezing point [°C]
0.91	2.75	6.21	3.85	14.3	8.9

### 2.2 Methods

#### 2.2.1 Synthesis of Copolymer Demulsifier P(AM‐EHMA‐VBS‐VP)

**Figure 1:**
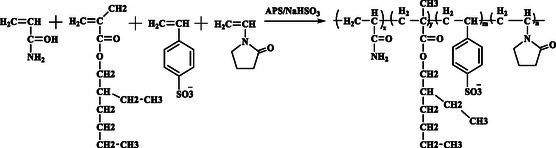
Schematic diagram of polymerization reaction.

The emulsion polymerization^[^ ^31^ ^]^ was carried out using a precisely controlled procedure. Initially, an aqueous surfactant solution was prepared by dissolving SDBS and NP‐10 in deionized water at a predetermined mass ratio. The monomer mixture, consisting of AM, EHMA, VBS, and VP in specific proportions, was then introduced into the surfactant solution. This mixture was emulsified using a T‐25 high‐speed homogenizer (IKA, Germany) operating at 20 000 rpm for 5 min at 30 °C to ensure uniform dispersion. Following emulsification, the polymerization was initiated by adding a redox initiator system comprising ammonium persulfate and sodium bisulfite in a 1:1 mass ratio. The mixture was vigorously stirred at 10 000 rpm for 3 min to ensure proper initiator distribution and the schematic diagram of polymerization reaction is shown in **Figure** **1**. To create an oxygen‐free environment crucial for radical polymerization, the system was purged with nitrogen for 5 min. The polymerization reaction was conducted at a controlled temperature range of 50–80 °C for a specified duration, resulting in the formation of a gel‐like polymer matrix. The obtained gel was subsequently processed through a series of purification steps: it was first cut into smaller pieces, then subjected to three cycles of ethanol washing and dehydration to remove unreacted monomers and impurities. Finally, the purified polymer was vacuum dried at 50 °C for 8 h and mechanically ground to pass through a 100‐mesh sieve, yielding a free‐flowing powdered demulsifier product ready for characterization and application testing.

#### 2.2.2 Characterization

The molecular structure of the synthesized P(AM‐EHMA‐VBS‐VP) copolymer was characterized using advanced spectroscopic techniques. Fourier‐transform infrared (FTIR) spectroscopy analysis was performed on a Nicolet 6700 spectrometer (Thermo Fisher Scientific, USA) employing the KBr pellet method, where the sample was thoroughly mixed with potassium bromide and compressed into transparent pellets for measurement. For nuclear magnetic resonance (NMR) analysis, the copolymer was dissolved in deuterium oxide (D_2_O), and examined using an AVANCE III‐400 spectrometer (Bruker, Germany) operating at 400 MHz for ^1^H NMR characterization. Thermal stability assessment was conducted using a Pyris‐1 Thermogravimetric Analyzer (PerkinElmer, USA). The analysis was performed under a controlled nitrogen atmosphere with a heating rate of 10 °C min^−1^, covering a temperature range from ambient to 800 °C to evaluate the copolymer's thermal degradation behavior.

#### 2.2.3 Evaluation of Demulsification Effect

The experimental procedure commenced with the preparation of simulated formation water, formulated to precisely match the ionic composition detailed in **Table** **2**. Following the industry specifications outlined in “General Technical Conditions for Crude Oil Demulsifiers (SYT 5280‐2018),”^[^ ^32^ ^]^ a W/O emulsion was systematically prepared. The emulsion system was established at a 1:1 volume ratio of crude oil to either simulated formation water or purified water. This mixture was subjected to continuous high‐shear mixing at 2500 rpm for 30 min using a precision homogenizer to ensure consistent emulsion formation. For demulsification evaluation, predetermined quantities of the synthesized demulsifier were introduced into the emulsion system and thoroughly mixed to ensure complete dissolution. The demulsification process was monitored under controlled conditions at 70 °C, with the dehydration rate quantified after a standardized period of 3 h. This procedure allowed for systematic assessment of the demulsifier's performance under conditions simulating actual field operations.

**Table 2:** The ion composition of formation water.

Ion composition [mg L^−1^]						
Cl^−^	Na^+^ + K^+^	Ca^2+^	Mg^2+^	Fe^3+^	HCO_3_^−^	TDS
17 125.26	5128.22	6781.26	2768.33	231.12	989.26	33 023.45

#### 2.2.4 Zeta Potential Measurement

The surface charge characteristics of aqueous droplets dispersed in the oil phase were quantitatively analyzed using a ZEN 3600 Zeta Potential Analyzer (Malvern Instruments, UK). This advanced laser‐based system enabled precise measurement of the zeta potential, a key indicator of droplet surface charge, which serves as a critical parameter for predicting interfacial phenomena and droplet coalescence behavior in emulsion systems. Measurements were conducted under controlled temperature conditions to ensure data reproducibility and relevance to actual operating conditions.

#### 2.2.5 Interface Tension Test

Interfacial tension analysis was conducted using an optical tensiometer (Attension Theta, Biolin Scientific, Sweden) equipped with a precision pendant drop module (PD‐200). The measurement protocol involved injecting crude oil through a specialized curved needle into quartz cuvettes containing demulsifier solutions of varying concentrations. This configuration enabled the formation of well‐defined pendant oil droplets at the needle tip, immersed in the aqueous phase. The instrument's advanced image analysis system continuously monitored and recorded the temporal evolution of interfacial tension with high spatial and temporal resolution, providing quantitative data on the dynamic interfacial behavior at the crude oil–water interface in the presence of the demulsifier.

#### 2.2.6 Interface Rheological Test

The interfacial dilational rheological properties, including both the elastic (*E*′) and viscous (*E*″) components of the complex dilational modulus (E), were systematically investigated using the oscillating drop module of an optical tensiometer (Attension Theta, Biolin Scientific, Sweden). Measurements were performed at the crude oil–water interface with varying concentrations of demulsifier solutions. The instrument's advanced oscillation mode enabled precise determination of the interfacial viscoelastic properties by subjecting the pendant oil droplet to controlled sinusoidal area perturbations while simultaneously monitoring the resulting interfacial tension response. This technique provided comprehensive characterization of the interfacial mechanical properties, crucial for understanding the demulsification mechanism at the molecular level.^[^ ^33^, ^34^ ^]^

(1) $$E=\frac{d\gamma }{dA/A}=\frac{d\gamma }{dlnA}$$

(2) E=|E|sinθ+i|E|cosθ=E′sinθ+E″cosθ

*γ* is the interface tension between crude oil and water solution, mN m^−1^; *A* is the interfacial area between crude oil and water solution, m^2^; and *θ* is the phase angle.

## 3 Results and Discussion

### 3.1 Optimization of Synthesis Conditions

#### 3.1.1 Optimization of Emulsifier Ratio and Dosage

**Figure 2:**
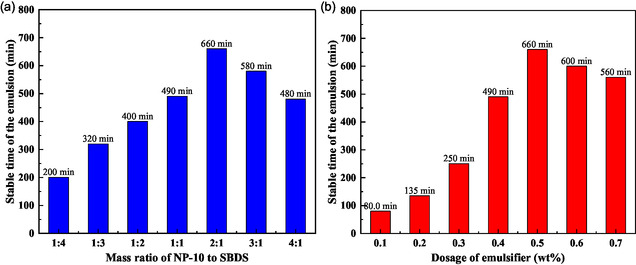
Optimization of mass ratio of emulsifiers and dosage in polymerization: a) Mass ratio of emulsifers; b) dosage of emulsifer.

The synthesis of polymer demulsifier P(AM‐EHMA‐VBS‐VP) was accomplished through emulsion polymerization,^[^ ^31^ ^]^ a method particularly suitable for incorporating the hydrophobic monomer EHMA. The success of emulsion polymerization critically depends on the formulation of an effective emulsifier system,^[^ ^31^, ^35^ ^]^ which must establish a stable W/O environment to facilitate proper polymerization kinetics. In this study, a binary emulsifier system comprising NP‐10 and SDBS was employed. The optimization process involved systematic evaluation of both the mass ratio between these surfactants and their total concentration, with the stability duration of the polymerization system serving as the primary evaluation metric. The polymerization was conducted with an equimolar ratio of the four monomers (AM:EHMA:VBS:VP = 1:1:1:1) at a fixed total monomer concentration of 30 wt%. **Figure** **2**a presents the optimization results for the NP‐10/SDBS mass ratio at a constant total emulsifier concentration of 0.5 wt%. The data revealed that a 2:1 mass ratio of NP‐10 to SDBS yielded optimal system stability, maintaining emulsion integrity for 660 min at ambient temperature without phase separation. This superior performance is attributed to the balanced hydrophilic–lipophilic properties achieved at this specific ratio.

Further optimization of emulsifier concentration, as shown in Figure 2b, demonstrated that system stability was highly concentration‐dependent. At lower concentrations (0.1–0.3 wt%), the polymerization system exhibited poor stability, while increasing the emulsifier content to 0.5 wt% significantly enhanced stability. However, exceeding this optimal concentration resulted in decreased stability, likely due to micelle formation and associated changes in interfacial properties.

Based on these systematic investigations, the optimal emulsifier formulation was determined to be a 2:1 mass ratio of NP‐10 to SDBS at a total concentration of 0.5 wt%. These conditions provided the most stable polymerization environment, ensuring successful synthesis of the target demulsifier.

#### 3.1.2 Optimization of Monomer Mass Ratio

The performance characteristics of the copolymer are fundamentally determined by its compositional parameters, particularly the mass ratio of constituent monomers. To systematically optimize the monomer composition, a four‐factor, four‐level orthogonal experimental design was implemented, considering the mass ratios of AM, EHMA, VBS, and VP as independent variables. The optimization process employed the crude oil dehydration rate as the primary evaluation metric, providing a direct measure of the copolymer's demulsification efficiency.

The experimental design matrix, presented in **Table** **3**, was constructed to efficiently explore the combinatorial space of monomer ratios while minimizing the number of required experiments. This orthogonal array approach allows for the simultaneous investigation of multiple factors and their interactions, providing comprehensive insights into the structure‐performance relationship of the copolymer.

**Table 3:** Factor and level design.

Factor\Level	AM	EHMA	VBS	VP
L1	1	1	1	1
L2	2	2	2	2
L3	3	3	3	3
L4	4	4	4	4

In the evaluation test, the polymerization temperature was 60 °C, the reaction time was 8 h, the total monomer concentration was 30 wt%, and the initiator dosage was 0.15 wt%. The dehydration rate of crude oil was tested by adding 300 mg L^−1^ of the product. The test results are shown in **Table** **4**.

**Table 4:** L16 (4^4^) orthogonal experimental results.

No.	AM	EHMA	VBS	VP	Dehydration rate [%]
1	1	1	1	1	53.1
2	1	2	2	2	68.5
3	1	3	3	3	78.1
4	1	4	4	4	85.2
5	2	1	2	3	41.1
6	2	2	1	4	50.2
7	2	3	4	1	88.2
8	2	4	3	2	89.7
9	3	1	3	4	55.1
10	3	2	4	3	65.2
11	3	3	1	2	70.2
12	3	4	2	1	88.3
13	4	1	4	2	50.1
14	4	2	3	1	52.3
15	4	3	2	4	67.2
16	4	4	1	3	68.3

The average and range analysis results are presented in **Table** **5**. According to the range analysis, the influence of the monomer ratio on the demulsification ability of the product is ranked as EHMA > VBS > AM > VP. This is because EHMA is the only lipophilic monomer, which plays a crucial role in the interfacial activity of the product. Meanwhile, based on the average value, the optimal monomer mass ratio is 1:4:4:1.

**Table 5:** Analysis of orthogonal experimental results.

Average/Range	Dehydration rate [%]			
	AM	EHMA	VBS	VP
*K*_1_	71.225	49.85	56.45	70.475
*K*_2_	67.3	59.05	66.275	69.625
*K*_3_	69.7	75.925	68.8	63.175
*K*_4_	59.475	82.875	72.175	64.425
*R*	11.75	33.025	15.725	7.3

#### 3.1.3 Optimization of Polymerization Conditions

**Figure 3:**
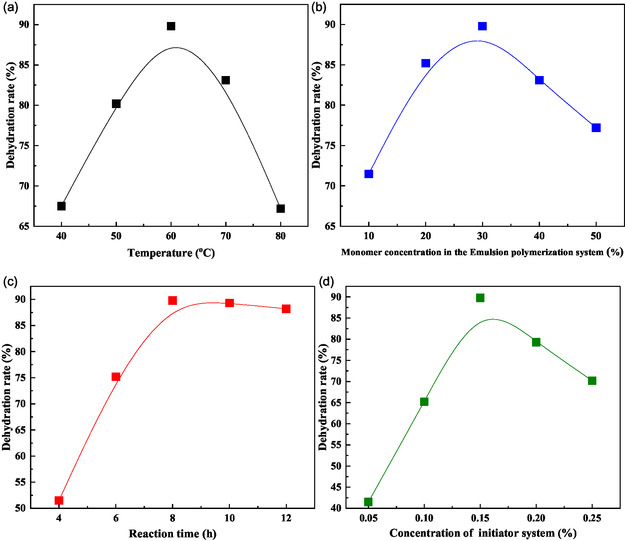
Optimization of reaction conditions: a) temperature, b) monomer concentration, c) reaction time, and d) concentration of initiator system.

Systematic optimization of the polymerization process was conducted using the established monomer ratio of 1:4:4:1 (AM:EHMA:VBS:VP), with the crude oil dehydration rate serving as the primary performance metric. The experimental results, presented in **Figure** **3**, provide comprehensive insights into the effects of key process parameters on demulsifier performance.

As shown in Figure 3a, the polymerization temperature significantly influenced the demulsifier's performance, with optimal dehydration capacity achieved at 60 °C. This temperature represents an ideal balance between reaction kinetics and molecular weight control. The concentration dependence study (Figure 3b) revealed that a monomer concentration of 30% yielded the most effective product, likely due to optimal viscosity and molecular weight characteristics achieved at this concentration.

Temporal analysis of the polymerization process (Figure 3c) demonstrated that product performance stabilized after 8 h of reaction time. Extended polymerization durations resulted in performance degradation, which can be attributed to broader molecular weight distributions and potential chain scission events. These structural changes negatively impact the demulsifier's interfacial migration capability and overall performance.

The initiator concentration study (Figure 3d) revealed a complex relationship between initiator loading and product performance. At 0.15% initiator concentration, the demulsifier exhibited maximum dehydration capacity. Lower initiator concentrations led to incomplete monomer conversion and heterogeneous molecular weight distributions, while higher concentrations caused excessive reaction rates, reduced molecular weights, and increased side reactions, all of which compromised demulsification efficiency.

Based on these systematic investigations, the optimal polymerization conditions for synthesizing P(AM‐EHMA‐VBS‐VP) demulsifier were determined to be: 1) Reaction temperature: 60 °C; 2) monomer concentration: 30% (w/w); 3) polymerization time: 8 h; and 4) initiator dosage: 0.15% (w/w).

These optimized conditions ensure the production of demulsifiers with well‐controlled molecular characteristics and superior performance in crude oil dehydration applications, while maintaining efficient process economics and reproducibility.

### 3.2 Demulsification Condition Optimization

**Figure 4:**
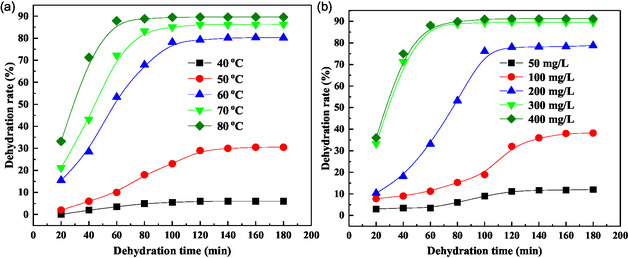
Optimization of demulsification condition: a) demulsifier dosage of 300 mg L^−1^ and b) demulsification temperature of 80 °C.

The efficiency of crude oil demulsification is governed by several critical operational parameters, with temperature and demulsifier dosage being particularly influential once the demulsifier type has been selected. Our systematic investigation of these parameters, presented in **Figure** **4**, provides valuable insights into their impact on dehydration efficiency.

#### 3.2.1 Temperature Optimization (Figure 4a)

The temperature‐dependent demulsification study, conducted at a fixed demulsifier concentration of 300 mg L^−1^, revealed significant thermal effects on dehydration kinetics. At 40 °C, the system exhibited poor performance with a dehydration rate below 10% after 3 h, indicating insufficient thermal energy for effective interfacial activity. Increasing the temperature to 50 °C improved the dehydration rate to 30.5%, reaching equilibrium within 2 h. This enhancement is attributed to intensified molecular motion and accelerated demulsifier migration to the oil–water interface.

The most pronounced improvement occurred at 80 °C, where the system achieved an 89.6% dehydration rate within 1 h. This optimal temperature represents a balance between enhanced molecular mobility and practical field application constraints. The thermal energy facilitates both demulsifier migration and droplet coalescence through increased collision frequency, while remaining within operational limits for field implementation.

#### 3.2.2 Dosage Optimization (Figure 4b)

At the optimized temperature of 80 °C, we investigated the dosage dependence of demulsification efficiency. Low concentrations (50 mg L^−1^) proved ineffective, while 100 mg L^−1^ provided limited improvement (<40% dehydration). A significant performance enhancement was observed at 200 mg L^−1^, achieving 78.8% dehydration within 100 min.

The optimal dosage was determined to be 300 mg L^−1^, yielding an 89.8% dehydration rate within 1 h. This concentration represents the saturation point for interfacial coverage, as evidenced by the negligible improvement observed at 400 mg L^−1^. The dosage optimization curve follows typical surfactant behavior, demonstrating a critical concentration threshold followed by a plateau region.

These findings establish 80 °C and 300 mg L^−1^ as the optimal operational parameters for field application of the P(AM‐EHMA‐VBS‐VP) demulsifier, providing both technical efficiency and economic viability.

### 3.3 Characterization

**Figure 5:**
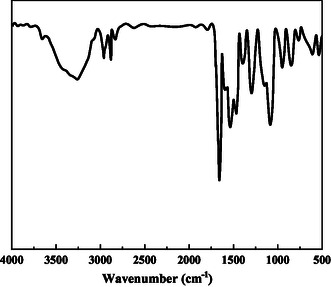
Infrared spectrum of *P* (AM‐EHMA‐VBS‐VP).

The structure of the demulsifier P(AM‐EHMA‐VBS‐VP) prepared by the optimized method was characterized by IR and 1H NMR. **Figure** **5** shows the infrared spectrum. The broad peak at 3261.5 cm^−^^1^ corresponds to the N—H stretching vibration peak, and 2967.1 cm^−^^1^ is the stretching vibration peak of —CH_3_, 2887.5 cm^−^^1^ is the stretching vibration peak of —CH_2_, 1780 cm^−^^1^ is the stretching vibration peak of C=O in the ester group, 1648 cm^−^^1^ is the stretching vibration peak of C=O in the amide, and 1598 cm^−^^1^ is the in‐plane bending vibration peak of N—H in the amide. 1068.9 cm^−^^1^ is the symmetric stretching vibration peak of the sulfonic acid group, 1156.7 cm^−^^1^ is the asymmetric stretching vibration peak of the sulfonic acid group, and 3066.9 cm^−^^1^ is the C—H deformation vibration peak of the benzene ring structure.

**Figure 6:**
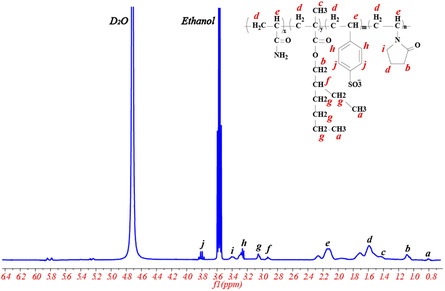
^1^H NMR spectrum of *P* (AM‐EHMA‐VBS‐VP).

The molecular structure of P(AM‐EHMA‐VBS‐VP) was also characterized by ^1^H NMR spectrum as shown in **Figure** **6**. The chemical shifts corresponding to resonances of H are as follows: *δ* 0.8 ppm (a) for the three H resonances of methyl (—CH2— **CH3** ) on the carbon chain structure; *δ* 1.09 ppm (b) for the two H resonances of methylene (—COO— **CH2** —or —CO— **CH2** —CH2—); *δ* 1.43 ppm (c) for the three H resonances of methyl (—C— **CH3** ); *δ* 1.59 ppm (d) for the two H resonances of methylene (—CH— **CH2** — or —C— **CH2** —); *δ* 2.13 ppm (e) for the H resonance of methine (— **CH** —CH2—); *δ* 2.93 ppm (f) for the H resonance of methine (—CH2— **CH** (CH2)2); *δ* 3.06 ppm (g) for the two H resonances of methylene (—CH— **CH2** — **CH2** — **CH2** —); *δ* 3.24 ppm (h) and *δ* 3.81 ppm (j) for the H resonances of benzene (—CH— **C6H4** —SO3); and δ 3.39 ppm (i) for the two H resonances of methylene (—N— **CH2** —CH2—).

### 3.4 Effect of Salinity on Demulsification

**Figure 7:**
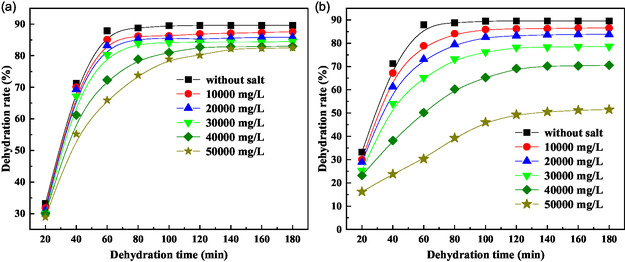
Effect of salt on dehydration performance a) NaCl and b) CaCl_2_.

The high salinity characteristic of formation of water in crude oil emulsions necessitates exceptional salt tolerance in demulsifiers for effective field applications. We systematically evaluated the salt resistance of P(AM‐EHMA‐VBS‐VP) by investigating its performance in the presence of sodium chloride (NaCl) and calcium chloride (CaCl_2_), with results presented in **Figure** **7**. All tests were conducted under optimized conditions (80 °C, 300 mg L^−1^ demulsifier concentration).

#### 3.4.1 Sodium Chloride Tolerance (Figure 7a)

The demulsifier demonstrated remarkable stability across a wide range of NaCl concentrations (0–30 000 mg L^−1^), maintaining consistent dehydration kinetics. This robust performance is attributed to the polymer's stable hydration layer and effective charge screening at moderate ionic strengths.^[^ ^13^, ^36^ ^]^ However, at elevated concentrations (40 000 mg L^−1^), a significant reduction in dehydration efficiency was observed. This performance degradation results from the disruption of the polymer's hydration shell and subsequent chain collapse, which impedes interfacial migration and reduces surface activity.

#### 3.4.2 Calcium Chloride Tolerance (Figure 7b)

The demulsifier exhibited greater sensitivity to CaCl_2_ concentrations, reflecting the stronger hydration layer disruption capability of divalent calcium ions compared to monovalent sodium ions.^[^ ^37^ ^]^ Despite this increased sensitivity, the polymer maintained effective performance up to 10 000 mg L^−1^ CaCl_2_, demonstrating its robust structural design.

These results establish that P(AM‐EHMA‐VBS‐VP) possesses excellent salt tolerance, with operational limits of 30 000 mg L^−1^ for NaCl and 10 000 mg L^−1^ for CaCl_2_. This performance profile meets or exceeds the requirements for most field applications, particularly in high‐salinity reservoirs. The demulsifier's salt resistance stems from its carefully engineered molecular structure, which combines hydrophobic moieties with ionic groups to maintain stability across a wide range of ionic strengths. The strongly hydrophilic sulfonate group in the VBS monomer significantly enhances both thermal stability and salt tolerance by effectively mitigating the salting‐out effect.^[^ ^38^ ^]^

### 3.5 Evaluation of the Demulsification Effect Based on Oilfield Conditions

To evaluate the relative performance of our synthesized demulsifier, we conducted comparative tests against SP‐DE, a polyether‐type demulsifier, is composed of polyoxyethylene‐polyoxypropylene octadecyl ether. The evaluation was performed using a (W/O) emulsion prepared with simulated formation water (composition detailed in Table 2) and crude oil, with both demulsifiers tested at 300 mg L^−1^ concentration.

**Figure 8:**
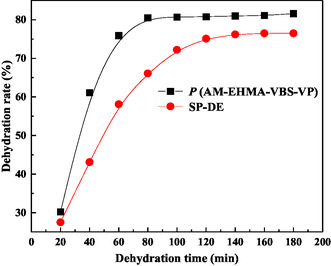
Dehydration performance of *P* (AM‐EHMA‐VBS‐VP) and the comparison sample under oil‐field operation conditions.

The temporal evolution of dehydration rates, presented in **Figure** **8**, reveals significant performance differences between the two demulsifiers. The P(AM‐EHMA‐VBS‐VP) copolymer demonstrated superior demulsification efficiency, achieving system equilibrium within 80 min with a maximum dehydration rate of 81.6%. In contrast, SP‐DE required 120 min to reach equilibrium, attaining a lower maximum dehydration rate of 76.5%.

This enhanced performance can be attributed to several structural advantages of P(AM‐EHMA‐VBS‐VP): 1) Tailored amphiphilic structure enabling faster interfacial migration; 2) optimal molecular weight distribution for effective film disruption; 3) enhanced thermal stability at the test temperature (80 °C); and 4) better compatibility with the high‐salinity environment.

The 6.6% improvement in maximum dehydration rate and 33% reduction in equilibrium time demonstrate the technical superiority of our synthesized demulsifier over the commercial benchmark. These results validate the effectiveness of our molecular design strategy and suggest significant potential for field applications.

### 3.6 Mechanism Investigation

The demulsification mechanism of P(AM‐EHMA‐VBS‐VP) was elucidated through a comprehensive suite of interfacial characterization techniques. Dynamic interfacial tension measurements^[^ ^33^, ^34^ ^]^ provided insights into the adsorption kinetics and interfacial activity, while zeta potential analysis revealed changes in the electrical double layer structure at the oil–water interface.^[^ ^26^ ^]^ Complementary interfacial dilational rheology experiments characterized the mechanical properties of the interfacial film, offering crucial information about film strength and viscoelastic behavior.^[^ ^23^ ^]^

This multi‐technique approach enabled a thorough understanding of the demulsification process at the molecular level, revealing how P(AM‐EHMA‐VBS‐VP) disrupts and destabilizes the protective interfacial film through a combination of: 1) Rapid interfacial adsorption and tension reduction; 2) effective charge neutralization of water droplets; and 3) modification of interfacial film rheological properties.

#### 3.6.1 Dynamic Interfacial Tension (IFT) Measurement

**Figure 9:**
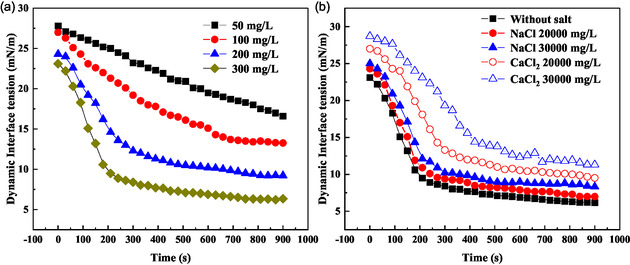
Effect of demulsifier dosage and salt on dynamic IFT: a) the effect of demulsifer dosage on dynamic IFT; b) the effect of salt type and dosage on dynamic IFT.

The interfacial behavior and demulsification mechanism of P(AM‐EHMA‐VBS‐VP) were systematically investigated through dynamic interfacial tension measurements under varying demulsifier concentrations and ionic conditions. **Figure** **9** presents the key findings from these interfacial characterization studies.

##### 3.6.1.1 Concentration‐Dependent Interfacial Activity (Figure 9a)

At lower concentrations (50–100 mg L^−1^), the demulsifier exhibited limited interfacial activity, characterized by slow tension reduction rates. This behavior reflects insufficient surface coverage and slow diffusion kinetics. However, at optimal concentrations (200–300 mg L^−1^), the dynamic interfacial tension profiles revealed three distinct stages: 1) Stage I: rapid adsorption phase: Characterized by a sharp decline in interfacial tension, this stage represents the fast diffusion and adsorption of demulsifier molecules at the oil–water interface. The rate of tension reduction is directly proportional to the demulsifier concentration; 2) Stage II: gradual organization phase: Marked by a slower tension decline, this phase corresponds to the structural reorganization of adsorbed molecules and continued migration of residual demulsifier to the interface; and 3) Stage III: equilibrium phase: The system reaches adsorption–desorption equilibrium, maintaining stable interfacial tension. Higher demulsifier concentrations accelerate the transition through these stages, demonstrating enhanced interfacial activity.

##### 3.6.1.2 Salt Effect on Interfacial Behavior (Figure 9b)

The influence of ionic strength on interfacial activity was investigated at the optimal demulsifier concentration (300 mg L^−1^). While all conditions maintained the three‐stage adsorption profile, indicating preserved salt tolerance, calcium chloride exhibited more pronounced effects than sodium chloride. This differential behavior stems from the stronger hydration layer disruption capability of Ca^2+^ ions compared to Na^+^ ions, leading to: 1) Partial polymer chain aggregation; 2) reduced interfacial migration efficiency; and 3) modified adsorption kinetics.

##### 3.6.1.3 Demulsification Mechanism

The comprehensive analysis reveals that P(AM‐EHMA‐VBS‐VP) achieves effective demulsification through a dual mechanism:^[^ ^23^ ^]^ 1) Interfacial activity: rapid adsorption and tension reduction at the oil–water interface and 2) film modification: structural reorganization of the interfacial boundary layer.

These synergistic effects destabilize the protective film around water droplets, facilitating coalescence and phase separation. The demulsifier's performance is maintained across a wide range of ionic strengths, demonstrating its suitability for field applications in diverse reservoir conditions.

#### 3.6.2 Interface Dilational Modulus Measurement

The interfacial behavior of polymer demulsifier P(AM‐EHMA‐VBS‐VP) was comprehensively characterized through interfacial dilational rheology measurements, providing crucial insights into its demulsification mechanism. The demulsifier's adsorption at the oil–water interface significantly modifies the interfacial rheological properties, reducing both the interfacial activity and mechanical strength of the interfacial film, thereby facilitating emulsion breakdown.

##### 3.6.2.1 Concentration‐Dependent Interfacial Rheology (Figure 10)

The effect of demulsifier concentration on interfacial properties was investigated through dilational modulus (*E*), elastic modulus (*E*′), and viscous modulus (*E*″) measurements. These parameters provide fundamental insights into the interfacial film's mechanical characteristics:^[^ ^33^, ^34^ ^]^ 1) dilational modulus (*E*): characterizes the interface's resistance to deformation; 2) elastic modulus (*E*′): reflects the interface's self‐healing capability; and 3) viscous modulus (*E*″): indicates the interface's resistance to structural changes.

**Figure 10:**
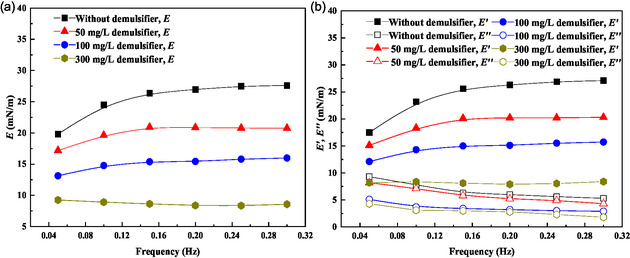
Effect of dosage on interface dilational modulus: a) *E*; b) *E*′; and *E*″.

As shown in **Figure** **10**a, increasing demulsifier concentration significantly reduces the dilational modulus across all frequencies, indicating weakened interfacial film strength. This reduction is particularly pronounced at lower frequencies, where the interface demonstrates higher elasticity in the absence of demulsifier. The frequency‐dependent behavior shifts from elastic‐dominated to viscous‐dominated characteristics with increasing demulsifier concentration.

Figure 10b reveals that both *E*′ and *E*″ decrease with higher demulsifier concentrations, with a more substantial reduction in *E*′. This indicates that the primary demulsification mechanism involves disruption of the interface's elastic recovery capability, making the film more susceptible to permanent deformation and rupture.

##### 3.6.2.2 Effect of salt on interface dilational modulus (Figure 11)

**Figure 11:**
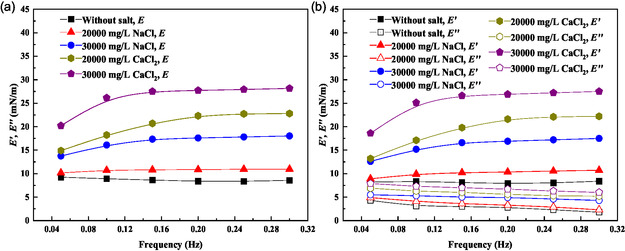
Effect of salt on interface dilational modulus: a) *E*; b) *E*′; and *E*″.

The impact of ionic strength on interfacial properties was evaluated at the optimal demulsifier concentration (300 mg L^−1^). **Figure** **11**a demonstrates that increasing salt concentration, particularly CaCl_2_, elevates the dilational modulus, with more pronounced effects at higher frequencies. This behavior results from: 1) Disruption of the demulsifier's hydration layer by ions; 2) reduced demulsifier adsorption at the interface; and 3) restoration of the interface's native elastic properties.

Figure 11b shows that higher ionic concentrations, especially Ca^2+^, maintain elevated values of both *E*′ and *E*″. The increased elastic modulus indicates enhanced interfacial film recovery capability, while the viscous modulus reflects greater structural stability. These changes are attributed to the competitive adsorption between ions and demulsifier molecules at the interface.

The comprehensive rheological analysis reveals that P(AM‐EHMA‐VBS‐VP) achieves effective demulsification through: 1) Interfacial activity reduction: lowering surface tension and modifying interfacial structure; 2) film weakening: reducing the interface's resistance to deformation; and 3) elasticity disruption: impairing the interface's self‐healing capability.

These effects collectively destabilize the protective film around water droplets, facilitating coalescence and phase separation. While ionic strength influences these mechanisms, the demulsifier maintains effective performance across a range of salt concentrations, demonstrating its suitability for field applications.

#### 3.6.3 Zeta Potential Test

**Figure 12:**
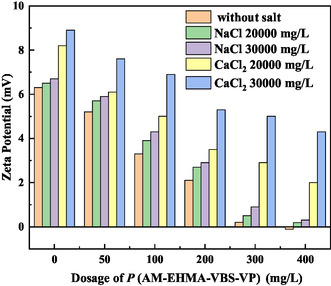
Effect of salt on zeta potential.

The electrokinetic properties of water droplets in crude oil emulsions were systematically investigated through zeta potential measurements, providing crucial insights into the electrostatic aspects of the demulsification process. As illustrated in **Figure** **12**, these measurements reveal significant effects of demulsifier concentration and ionic environment on emulsion stability.

The zeta potential analysis demonstrates an inverse relationship between demulsifier concentration and droplet surface charge. This phenomenon results from the effective charge shielding provided by P(AM‐EHMA‐VBS‐VP) molecules at the oil–water interface. The adsorption of demulsifier molecules neutralizes the native charges on crude oil components, reducing the electrostatic repulsion between droplets and facilitating coalescence.^[^ ^23^, ^24^, ^25^, ^26^ ^]^

The ionic environment significantly influences this electrokinetic behavior: 1) Sodium ions (Na^+^) moderately increase the zeta potential through partial charge screening and 2) calcium ions (Ca^2+^) exhibit more pronounced effects due to: a) stronger charge neutralization capability, b) competitive adsorption at the interface, and c) disruption of demulsifier hydration layers.

These ionic effects create a complex relationship between emulsion stability and water chemistry: 1) higher ionic concentrations, particularly of Ca^2+^, maintain elevated zeta potentials; 2) increased electrostatic repulsion between droplets enhances emulsion stability; and 3) demulsifier effectiveness is consequently reduced in high‐salinity environments.

The combined analysis of zeta potential measurements and interfacial characterization reveals that P(AM‐EHMA‐VBS‐VP) achieves effective demulsification through a dual mechanism: 1) electrostatic effects: charge neutralization and reduction of inter‐droplet repulsion and 2) mechanical effects: interfacial film weakening and structural modification.

These findings demonstrate the importance of both electrostatic and mechanical factors in emulsion stability and provide a comprehensive understanding of the demulsification process at the molecular level.

## 4 Conclusion

This study successfully developed a novel copolymer demulsifier, P(AM‐EHMA‐VBS‐VP), through optimized emulsion polymerization. Systematic optimization using orthogonal array design and single‐factor experiments established the following optimal synthesis parameters: monomer mass ratio, emulsifier system composition, concentration, reaction temperature (60 °C), polymerization duration (8 h), monomer concentration (30 wt%), and initiator dosage (0.15 wt%). Structural characterization through FT‐IR and ^1^H NMR spectroscopy confirmed the successful synthesis of the target molecular architecture.

Performance evaluation under simulated field conditions demonstrated exceptional demulsification efficiency, with an optimal dosage of 300 mg L^−1^ and operational temperature of 80 °C. The demulsifier exhibited remarkable salt tolerance, particularly against sodium chloride (up to 30 000 mg L^−1^), though calcium chloride showed more pronounced inhibitory effects at lower concentrations (10 000 mg L^−1^). Field‐relevant testing using Yumen Oilfield formation water (total salinity: 33 023.45 mg L^−1^) yielded an 81.6% dehydration rate, surpassing the performance of commercial fatty alcohol polyoxyethylene ether demulsifier SP‐DE (76.5%).

Mechanistic investigations through dynamic interfacial tension measurements, interfacial dilational rheology, and zeta potential analysis revealed a multifaceted demulsification mechanism: 1) interfacial activity: significant reduction of interfacial tension through rapid adsorption; 2) film modification: decrease in interfacial viscosity and mechanical strength; 3) structural weakening: reduction of E, *E*′, and *E*″; and 4) electrostatic effects: charge shielding leading to reduced zeta potential and enhanced droplet coalescence.

The study also elucidated the inhibitory effects of inorganic salts, particularly calcium chloride, which disrupts the demulsifier's hydration layer and impedes interfacial adsorption. These findings provide valuable insights for optimizing demulsifier performance in high‐salinity environments.

The developed P(AM‐EHMA‐VBS‐VP) demulsifier demonstrates superior performance characteristics compared to conventional products, offering significant potential for enhanced oil–water separation in challenging field conditions. Future research directions include field trials and optimization for specific reservoir conditions to facilitate commercial implementation.

## Conflict of Interest

The authors declare no conflict of interest.

## Data Availability

Research data are not shared.
